# Society Proceedings

**Published:** 1902-05

**Authors:** 


### SOCIETY PROCEEDINGS.

### ILLINOIS STATE VETERINARY MEDICAL ASSOCIATION.

THE twentieth semi-annual meeting was called to order by President Joseph Hughes at the Hotel Fey, Peoria, Ill., February 19, 1902. The following members were present:

Drs. J. T. Nattress, Delavan; Albert Babb, Springfield; L. C. Tiffany, Springfield; D. E. Kinsella, Chillicothe; F. H. Ames, Canton; M. A. Storry, Bradford; N. I. Stringer, Watseka; T. J. Gunning, Neponset; H. A. Pressler, Fairbury; C. D. Hartman, Peoria; John Scott, Peoria; E. J. List, Havana; W. H. Welch, Lexington; A. C. Worms, Chicago; E. L. Quitman, Chicago; Joseph Hughes, Chicago; Jas. Smellie, Eureka.

Visitors: Drs. M. C. Eckley, Galesburg; E. D. Yrion, Elmwood; Jas. Wood, Pekin, and Mr. Louie Riesz, representing the drug firm of Sutliff & Case, of Peoria.

The minutes of the last meeting were read and approved.

The following applications were received and on motion were elected to membership: Dr. L. C. Tiffany; vouchers, Drs. John Scott and T. J. Gunning. Dr. D. E. Kinsella; vouchers, Drs. John Scott and Jos. Hughes.

The bills for Secretary's fee and stationery for $40.50 were audited and ordered paid. Receipts were $13.

Report of Treasurer showed balance on hand of $42.85.

Mr. Riesz, on behalf of Sutliff & Case, extended an invitation to visit their elegant establishment, which was accepted, and the meeting adjourned until 1 P.M.

The Association reconvened at 1 P.M.

Dr. Albert Babb read his paper on "The Business Relations of the Veterinarian."

Discussed by Drs. Quitman, Stringer, Worms, Scott, and Hughes.

Dr. H. A. Pressler read his paper, "A Peculiar Complication of Strangles."

Discussed by Drs. Quitman, Stringer, List, and Hughes.

F. H. Ames read reports of cases:

1. A peculiar growth on stifle of cow.
2. Case of retained fœtus in womb.
3. Scirrhus cord.

Discussed by Drs. Stringer, Gunning, Nattress, and Welch.

Dr. Nattress read report of cases as follows:

Trephining both sinuses.

Amputation of rectum.

Remarkable recovery of dog which had its leg nearly cut off.

Discussed by Drs. List, Quitman, and Worms.

Dr. Tiffany gave a short talk on the so-called "Cornstalk Disease."

The following resolutions were offered and adopted unanimously:

*Resolved*, That the Illinois State Veterinary Medical Association does hereby protest against the custom compelling graduates of recognized threeyear schools to pass the examination before the State Board of Veterinary Examiners.

*Resolved*, That the Illinois State Veterinary Medical Association does hereby protest against any professor or instructor connected with any veterinary college in the State serving on the State Board of Veterinary Examiners.

The By-laws, Article I. Section 2, were changed so as to read December instead of November.

Adjourned, to meet at Chicago in December at call of President.

W. H. Welch,
Secretary.

### OFFICIAL REPORT OF THE PROCEEDINGS OF THE FOURTEENTH ANNUAL MEETING OF THE IOWA STATE VETERINARY MEDICAL ASSOCIATION.

*Held at Des Moines, Iowa, February* 11 *and* 12, 1902.

(Concluded from page 258.)

### Report of Committee on Sanitation.

By way of introduction or explanation of this report, its chairman wishes to state that no effort has been made to advance any new or original ideas on this subject, but that the committee has gathered from contemporary veterinary literature and personal observation a few of what seemed to be the most pertinent facts and theories as applied to present local conditions.

The work has all been done in the last three weeks. It was arranged, at the suggestion of your worthy Secretary, to give Dr. Miller the ground covering State and municipal meat-inspection. His plan of work was to send out about twenty letters to each of the cities and to some of the more important towns of our State, with the following questions:

1. How many slaughtering establishments have you, not including those having Federal Government inspection?
2. How many are within the city limits?
3. Give approximate number of animals slaughtered annually.
4. Are all of these animals inspected before and at the time of slaughter by a city official?
5. What are the qualifications of the inspector?
6. What disposition is made of the condemned parts and carcasses?

He states that only a few replies have been received, and that they were very slow in coming in—the usual fate of such inquiries, so far as I am able to determine. In fact, there is no means whereby any statistics of this nature can be gathered. His report is inserted verbatim in the body of this report. To Dr. Gillian was assigned the task of reporting on State and municipal milk-inspection. His report was placed in my hands yesterday, February 10th, and will be read as given. Your chairman then had left for his consideration the State control of contagious diseases and the laws regulating this work. A word in passing may be devoted to a consideration of the relations these sustain, or should sustain, to those of our sister States and those of the Federal Government. We have no instantaneous, sure-shot, cut-and-dried remedy to recommend for the cure of all the ills that ignorance and contempt of sanitary law engender. We are also willing to concede in the broad field of sanitary science the relative importance of the work of the regular physician, the sanitary engineer, the bacteriologist, the chemist, and all others engaged in any branch of the complicated work of the modern sanitarian, even to our friend the original surgeon, the barber, in his legal attempts to assure us that he is shaving us with a clean razor. Certain it is that the keystone of progress in modern medicine is prevention rather than cure. We believe that all sanitary work should be concentrated into national, State, and local Boards of Health which work together harmoniously, and that this work should be directed by these boards; also, that any State or municipal sanitary laws, rules, or regulations should as far as possible supplement or augment the Federal system already established, and should not entail any unnecessary hardships or inconveniences upon those directly concerned.

It is almost self-evident that no State, with our modern methods of commercial activity and free exchange of all commodities, can of itself protect all its interests from invasion from without and from enemies within without the help of the General Government. And, indeed, after conceding all that the General Government has done or is willing and able to do, there will still be enough left to keep all trained sanitarians busy at work for the remainder of our natural lives at least.

### Municipal Meat-inspection.

To ascertain what is being done in municipal meat-inspection, your committee sent out a letter of inquiry to the Board of Health of each city and some of the more important towns of our State. Only five or six replies have been received. These are, however, from places that may be considered a fair average of the whole, as they are among the representative cities of the State.

All have slaughtering-houses, varying in number from two to five, some of which are situated within the city limits. We have not been able to ascertain the number of animals slaughtered annually, but it is safe to state that the aggregate number would amount to many thousands, yet the answers received would indicate that none of these animals is inspected either before or at the time of slaughter. That there are some diseased animals among all of these, and parts of carcasses that should be condemned, there is no doubt; but nobody has any knowledge of what disposition is made of them or to what use they are put. We can imagine, however!

One reply indicated that the subject had been discussed, but that no action had been taken.

The great majority of our people are not aware of the necessity of this inspection. The danger of a contaminated meat-supply is best known to veterinarians, and it is incumbent upon us to make known this danger to others and do all we can to bring about an adequate system of inspection. To this end your committee believes that veterinarians should be made members of Boards of Health whenever practical, and we would recommend that articles be published from time to time in our newspapers bearing upon the different phases of this subject; that each municipal council should pass an ordinance bringing each slaughter-house in the vicinity under municipal control, requiring them to do all their slaughtering at one abattoir, and that at a time when the council can best provide a qualified inspector, chosen by them, and to whom he must render a regular report.

### Dairy and Milk-inspection.

It has been requested by the chairman of the Sanitation Committee that I prepare a report on "Dairy and Milk-inspection," and setting forth what it should be. The inspection of the dairies and milk in my part of the State has received no attention at all in regard to sanitary conditions, and the dairymen conduct their dairy business in a way that is satisfactory to themselves, taking no thought of the consumers of the milk.

The dairy and its product should receive our best attention, as there is no one article of food in such universal use as is milk. Possessing as it does all the elements necessary for primary growth, it is the initial and only food for the newly born, and continues as such for months. Milk will receive and convey the odor of its surroundings. The unpleasant flavor of certain vegetables and sour or fermented foods which the cow may eat is readily detected in milk, and of all food that reaches our table none affords a more genial habitation for nearly every form of bacterium than milk. Not a year passes but that we find living proof of communicability of disease of various kinds through the medium of milk.

Milk is part of the cow, and is therefore animal matter. If the cow is tuberculous her milk is part of a diseased cow, and no matter how much it is boiled it still remains part of a diseased cow, and should not be used for human food. Now, while we are trying to protect the public against the use of this diseased milk we should also protect the dairyman and warn him against buying well-bred cows to increase the richness of his milk simply because he can buy them cheap, lest he introduce some disease with them into his herd. I doubt if anyone who has not been actually engaged in the inspection of dairy stables can appreciate the condition in which cows are sometimes found. Inflammation of the udder is a very common affection, and in this case the secretions from the diseased organ pass away with the milk. In some cases there is tuberculosis of the organ, when, of course, the condition is even more serious. We may readily see that one or two diseased cows can contaminate the milk of a whole herd and render it unfit for use. There are many cases on record in which there is undeniable proof of the spread of typhoid fever, diphtheria, and many other forms of disease through the medium of milk.

The dairy industry is getting to be a very common one in our country, especially in the larger cities, and we find only a small portion of the dairies under sanitary inspection. In this field I believe we can do a great work for humanity. We should impress upon the dairyman the criminality of selling milk from diseased cows, and induce him to apply sanitary principles to his dairy, such as thorough cleanliness, light, good ventilation, pure water-supply, and good, wholesome food.

We should be in touch with our health officers in our locality, working together in one common cause to maintain the health of the community. It is along this line of work that the veterinarian can make for himself a reputation worthy of his calling. The only way these things can be accomplished is by legislation.

It now remains with this Association to use every means in their power to see that some proper law is passed by our Legislature requiring that all dairies should be under strict inspection.

### State Control of Contagious Diseases.

Regarding contagious animal diseases, our State this year seems to be remarkably fortunate, so far as I am able to discover. The one disease that overshadows all others in its peculiarly slow, insidious, and almost imperceptible, but none the less fatal, progress marches on almost unhindered and, I might say, by the large majority yet unknown. Tuberculosis seems still to elude all sanitary attempts of States and nations to satisfactorily control its ravages among human beings or their animal servants. Our State, up to within a few years, has been comparatively free from its grasp on our domestic animals; but we are fast becoming, as it were, a dumping ground of high-bred infected stock from other States used in our higher-bred herds for the supposed purpose of improvement. From these it is carried to all classes of stock and to all localities into which they may happen to be taken. Perhaps the warning recently given by the Secretary of Agriculture against the importation of high-bred stock, together with the tuberculin test imposed on all imported stock under his direction, will do much to educate those concerned as to the nature and prevalence of the disease.

The stock owner should remember that, even though there is at present some reason to believe or rather to hope that the principal source of infection for human beings is other human beings, and for animals is other animals, an ounce of prevention in either case is worth many pounds of cure. The modern history of our knowledge of this disease is largely a recital of the achievements of Prof. Koch and his influence on the work of other modern investigators, and may be divided into three chief epochs. the first dates from his modest announcement, in 1882, that he had traced tuberculosis to the presence of a bacterial parasite. Seldom, if ever, had any medical discovery been received immediately with such intense interest, and yet the ground at that time was thoroughly broken for the reception of this seed, which immediately took root and thrived like the proverbial green bay-tree. This discovery gave a wonderful impetus to the work of modern pathology.

The second epoch dates from the somewhat more sensational announcement of the secular press, in 1890, of his discovery of a cure for this disease in tuberculin. Prof. Koch was then in the employ of his government at a salary of $7000 per annum, besides a pension, had one of the finest laboratories for bacteriological research in the world, and was assisted by a well-trained and well-paid corps of assistants. Though tuberculin did not fulfil its promise as a curative agent, it has paid its cost many times over as a diagnostic agent.

The third epoch was ushered in last summer at the British Congress on Tuberculosis by the widely heralded announcement by this same Prof. Koch, that human tuberculosis cannot be transmitted to cattle, and very strongly intimating the reverse conclusion, that animal tuberculosis is very seldom if ever transmitted to the human being. This latter part especially has raised a storm of protest among investigators the world over, and even though the specific contention may be proved beyond a doubt untrue, it will have accomplished a grand result by spurring on its final settlement.

In reviewing the legislation by the different States and Territories on this subject as compiled by the Department of Agriculture in Bulletin No. 28 we find forty-nine different laws or absences of law enumerated regulating or intended to regulate this malady in the different States and Territories.

The laws of our own State make no specific reference to tuberculosis. The only mention is in Rule 12 of the rules and regulations of the State Board of Health, which reads: "In suspected cases of bovine tuberculosis the tuberculin test shall be recognized as a valuable diagnostic." This is excellent as far as it goes, but whether this declaration is all the law in the case will warrant or the expediency of circumstances permit, we are without the necessary data to judge. The State Veterinarian, by virtue of his office as a member of the State Board of Health, is looked upon as the State veterinary sanitarian, and if the law as it now exists is too vague and indefinite to allow of anything more explicit he should have the hearty and concerted support of every graduated veterinarian and his friends in this State to bring about the passage of a law or the adoption of rules and regulations that his experience will have shown to be practical.

The next most insidious disease which the sanitarian of this State has to deal with is, perhaps, glanders. We believe this disease to be more generally understood and feared by the community at large than is tuberculosis.

Anthrax, so far as I have been able to determine, has not gained any permanent foothold in our State as it has in certain States farther South on the Mississippi. There is, however, always a possibility of its invading our territory.

Sporadic outbreaks of rabies and other infectious diseases have been discovered within our borders, and were promptly and efficiently handled by the State Veterinarian and his assistants.

Hog cholera and swine plague for some reason or other seem to have spent their fury, and the hog has seemed to enjoy extraordinarily good health the past season. How much this may be due to an enforced economy of feeding, on account of the high prices of grain, I shall leave you and the producer to conjecture. Certainly it seems that a large part of the annual loss from this source could be obviated by intelligent sanitary measures. It will certainly not be contended that this improvement has been brought about by the strict enforcement of the law against buying, selling, or giving away the carcasses of hogs dead from this disease, or against the transportation of infected hogs over the public highways.

It would be highly interesting and instructive to know how much the prevalence of this disease has been lessened by the general early sale of all suspicious hogs for immediate slaughter, and the consequent removal of these centres of infection with as little delay as possible, although this is in direct opposition to the intention of the law. This law, however, has been of inestimable value in fixing a large per cent. of the loss on the original owner of this stock by his willingness to refund money paid for diseased stock rather than stand for prosecution under the statute. In passing I might remark that I do not know of a better or more economical plan of rendering innocuous any carcass or part of a carcass affected with any known contagious disease than by the method of tanking now employed around any large slaughter-house; and this one point alone would render desirable the consolidation of the little country slaughter-houses into one well-equipped house, if for no other reason. In this way the different centres of infection operating through the feeding of offal in a raw state to other stock would be eradicated.

As to the transportation over our railroads of stock intended for breeding or feeding, we believe that this can be done only in freshly disinfected cars. The stock should be loaded at temporary movable chutes that have likewise been recently disinfected. The same precautions should be observed in unloading.

Regarding sheep scab, I wonder how many of our members know of the provision of our code for the handling of this malady, and whether it has ever been enforced; and, if so, why it cannot be made to come under the jurisdiction of the State Veterinarian.

Finally, regarding legislation to right some of these matters, your chairman recommends the concentration of our energies through our Committee on Legislation, with the object of procuring legislation that will ultimately result in compulsory State meat-inspection under the supervision of the State Veterinarian or the State Board of Health, with the State Veterinarian as its executive officer. At present the law should not be mandatory, but should simply enable cities, towns, or townships to regulate their local affairs through their local boards, subject to certain restrictions of the State board. There ought also to be some scheme devised whereby vital animal statistics and statistics of country slaughter-houses not under inspection could be obtained and classified under this same authority. In a word, publicity and a general enlightenment of ourselves and the public is the key to the adjustment of these problems. The municipal slaughter-house combined with a system of insurance of clinically sound animals under the supervision of well-trained, honest, courteous, discreet inspectors will solve the problem and lead to the establishment of a system for the detection and prevention of most of our contagious diseases.

As we said before, there is nothing new or original in this report. We have in our small way gathered a few ideas from the various sources at our command which may be termed sprigs of promise that, with a little mixture of technical ability and common-sense, may be engrafted upon the branches of the hardy old tree of existing conditions without any fear of injuring its vitality and with some hope of improving the quantity and quality of its fruit and the beauty of its foliage.

Respectfully submitted,
T. A. Shipley,
J. Miller,
H. M. Gillian,
Committee.

On motion, the report was received.

Dr. S. H. Kingery then presented an extemporaneous report on "Ravages of Strongylus Tetracanthus."

The report of Dr. E. G. Marten on "Urethral Calculus" was in his absence read by the Secretary.[1]

[1] See Journal for April, 1902, p. 205.

The report of Dr. G. P. Statter on "A Cow Case" was in his absence read by the Secretary.[1]

In the absence of Dr. J. Thomsen his report on "Open Joint" was read by Dr. Scott.[2]

Dr. C. C. Lyford, of Minneapolis, Minn., was introduced, and spoke on the subject of the meeting of the Veterinary Medical Association, which takes place at Minneapolis in September next. He gave all graduated veterinarians a cordial invitation to attend the meeting, assist it by their presence, and receive its benefits. He said that arrangements had been made to entertain the members and visitors and their ladies, and that the ladies would be admitted to the banquet.

Dr. S. D. Brimhall, also of Minneapolis, Minn., was introduced, and after extending an invitation to the graduated veterinarians of Iowa to attend the sessions and social functions of the meeting of the American Veterinary Medical Association, next September, he gave a short talk on the veterinary sanitary laws of Minnesota. As Dr. Brimhall is veterinary officer of the State Board of Health of Minnesota, and is charged with the administration of the State veterinary laws, this talk from him was very much appreciated.

Dr. Kingery moved that a committee of three be appointed by the President for the purpose of furthering the interests of the meeting of the American Veterinary Medical Association, to be held at Minneapolis next September. Seconded, put to vote, and carried.

The President appointed the following committee; Dr. J. I. Gibson, Dr. John J. Repp, Dr. W. A. Heck.

In the absence of Dr. L. U. Shipley, his report on "Typhoid Fever in a Horse" was read by Dr. Gibson.[3]

In the absence of Dr. Wm. Drinkwater, his paper, "Parturition Cases," was read by the Secretary.[4]

On vote of the Association, Dr. C. C. Lyford and Dr. S. D. Brimhall were elected to honorary membership in the Association.

Dr. H. E. Talbot, Committee on Clinics, announced the operations for which he had arranged, and urged a full attendance.

Dr. C. E. Stewart moved to adjourn until 8 o'clock the following morning, to meet at Dr. Talbot's infirmary. Seconded, put to vote, and carried.

### SECOND DAY—*February 12th.*

*Morning Session.* The forenoon was devoted to a clinic held at Dr. Talbot's infirmary. The operations were as follows:

Castration of double cryptorchid, by Dr. C. E. Stewart; castration of single cryptorchid, by Dr. P. Malcolm; median neurectomy for ringbone, by Dr. D. H. Miller and Dr. P. Malcolm; removal of tumor from sow's udder, by Dr. W. A. Heck; arytenoidectomy for roaring, by Dr. J. H. McNeall; peroneal tenotomy for stringhalt, by Dr. W. A. Heck and Dr. H. E. Talbot.

Dr. Talbot had charge of the clinic, and the arrangements were very satisfactory. The operations were well performed, and the clinic proved very interesting and instructive.

[1] See JOURNAL for April, 1902, p. 207.

[2] Ibid., p. 208.

[3] Ibid., p. 210.

[4] Ibid., p. 211.

*Afternoon Session.* Dr. D. E. Baughman presented his paper on "Rabies."[1]

Dr. George M. Walrod read his paper on "Amputation of a Bull's Penis."[2]

Dr. P. Malcolm presented his paper on "Abortion in Cows."[3]

Dr. H. L. Stewart read his paper on "Cæsarean Section."[4]

Dr. S. T. Miller then presented his paper on "External Ulcerative Ano-vulvitis."[5] He said that the name had been suggested to him by Dr. Repp as a substitute for the term "infectious ulcer of the vulva," a name which had been applied to this disease, but did not seem appropriate.

In the temporary absence of Dr. H. E. Talbot from the convention hall, his paper on "The Trials of the Veterinary Examining Board"[6] was read by the Secretary.

Dr. Gibson moved that the Secretary be authorized to edit the proceedings of this meeting and to have them printed in the *American Veterinary Review* and THE JOURNAL OF COMPARATIVE MEDICINE AND VETERINARY ARCHIVES, if the editors could be induced to publish them, or to have them printed in pamphlet form if it could be done free of cost by means of advertisements; the Secretary to use his discretion in making choice between the two methods of publication and to receive the sum of twenty dollars for his services in this connection. Seconded, voted upon, and carried.

The Secretary moved that the next meeting be held at Cedar Rapids. Seconded, and after some discussion voted upon and carried.

On motion, it was decided to hold the next meeting between December 1, 1902, and March 1, 1903, the exact time to be decided upon by the President and Secretary.

The Committee on Resolutions then reported as follows:

MR. PRESIDENT AND MEMBERS: We your Committee on Resolutions beg leave to report as follows:

*Resolved*, That it is the sense of this Association that the State Legislature should at its present session be very generous in the matter of granting the appropriation asked for and so greatly needed for the use of the veterinary section of the Experiment Station at Ames, so that comprehensive investigation may be made in reference to the nature, prevention, and treatment of the many diseases of our domestic animals which are so imperfectly understood and which cause such extensive losses, with a view to the prevention of these losses; also, that the most liberal spirit should control the Legislature in granting the appropriations for the other branches of work in the Experiment Station and to the State College for support and buildings.

*Resolved*, That we believe that it is requisite for the proper advancement of the Division of Veterinary Medicine of Iowa State College that its management be put into the hands of a veterinarian; therefore, we would respectfully request the Board of Trustees of that institution to place a veterinarian in the position of dean of that division of the college.

*Resolved*, That this Association gives its hearty support and assistance in every way possible to the State Board of Veterinary Medical Examiners, in order that the best results may accrue to our profession now and in future years.

[1] See JOURNAL for April, 1902, p. 214. [2] See p. 302. [3] See p. 280.

*Resolved*, That we consider the Bang method of suppression of tuberculosis of cattle to be the best method available, and that we commend it to the cattle breeders of the State.

*Resolved*, That this Association believes that there should be a complete revision of the laws of our State relating to veterinary sanitation and the powers and duties of the State Veterinarian and his assistants. Especially do we believe that all the laws in reference to sheep scab should be repealed and more effective laws enacted in their stead, putting the control of this disease into the hands of the State Veterinary Department and providing sufficient funds to carry on the work.

*Resolved*, That it is the opinion of this Association that Section 5012 of the code should be amended by striking out the following words, to wit: "knowingly" in the second and the third line, and "knowing the same to be" in the fifth line; and, further, that Section 5013 should be amended by striking out the following words, to wit: "knowingly" in the first line, "nasal gleet" in the second line, "button" in the third line, and "knowing the same to be" in the sixth line; and, further, that Section 5014 should be amended by striking out the following words, to wit: "nasal gleet" and "button" in the second line.

*Resolved*, That it is the sense of this Association that a competent veterinarian should be chosen by the Superintendent of the Horse Department of the State Fair to pass upon the soundness and freedom from hereditary unsoundness of all horses exhibited at our State Fair; be it further

*Resolved*, That a copy of this resolution be sent to the Secretary of the State Board of Agriculture and to the Superintendent of the Horse Department of the State Fair.

*Resolved*, That expert judging of horses, both as to soundness and individual excellence, be made a feature of our next clinic; and be it further

*Resolved*, That the Committee of Clinic be hereby requested to arrange for the presence for the above purpose of at least two classes of draft horses and two classes of road or carriage horses.

*Resolved*, That we express our strong appreciation of the earnest efforts of the late Dr. Rush Shippen Huidekoper in behalf of the veterinary profession in America as author, editor, teacher, and practitioner, and especially his efforts in the interest of army legislation; that by his death we have lost one of our most powerful supporters and coworkers, and that we extend to Mrs. Huidekoper our tenderest sympathy for her in her great personal loss.

*Resolved*, That we extend our thanks to the management of the Savery Hotel for placing at our disposal a convention hall free of charge, and to the newspapers of Des Moines for their courteous treatment.

George A. Scott,
J. I. Gibson,
F. J. Neiman,
Committee.

The report of the Committee on Resolutions was, on motion, unanimously adopted.

The election of officers resulted as follows:

President: Dr. J. I. Gibson, Denison.

First Vice-President: Dr. W. A. Heck, Maquoketa.

Second Vice-President: Dr. T. A. Shipley, Cedar Rapids.

Secretary and Treasurer: Dr. John J. Repp, Ames.

Board of Censors: Dr. S. K. Hazlet, Oelwein; Dr. George A. Scott, Independence; Dr. W. H. Austin, Newton.

The appointment of committees was deferred.

Dr. W. A. Heck offered special resolutions, as follows:

*Resolved*, That inasmuch as the offering of unsound stallions to the public for breeding purposes is a menace to the best interests of the horse breeders of the State, we recommend the enactment by the Legislature at its present session of a law requiring the licensing of all stallions used for public service as sires.

*Resolved*, That we extend to Dr. H. E. Talbot a vote of thanks for providing such an extensive clinic for our benefit, and that the usual appropriation be made to defray the expense in connection therewith.

On motion, these resolutions were adopted.

The Committee on Resolution on Dr. John E. Brown reported the following resolution:

*Resolved*, That we extend to Dr. John E. Brown, our former Secretary, our hearty good-will, an expression of our high appreciation of his generous efforts through so many years in behalf of our Association, and our best wishes for the health, happiness, and success of himself and his family in their new Southern home.

J. I. GIBSON,
GEORGE A. SCOTT,
JOHN J. REPP,
Committee.

On motion, it was unanimously adopted.

The Secretary moved that the President appoint a Committee on Legislation. Seconded, voted upon, and carried.

The Committee on Disease and Treatment submitted a report as follows:

### REPORT OF COMMITTEE ON DISEASE AND TREATMENT.

My report on disease and treatment must be brief. The members of the Association have been somewhat negligent in reporting for this committee. Abundant material could be offered if members would take notes on cases, observing the new phases of disease, and its prevention and treatment.

In this State the usual diseases have appeared. Tuberculosis is on the increase. It has been noticed, however, that the most intelligent, active, and successful farmers are now beginning to view tuberculin as a valuable aid to diagnosis. They take more kindly to the tuberculin test, and feel that it is the only method by which a check can be put upon the course of this scourge. Actinomycosis is prevalent. Treatment with potassium iodide in the early stages is most gratifying, and in the later stages excision of growths and bone if necessary is of much benefit, and cures a large majority of cases. Bichromate of potassium in 20 per cent. solution has proved valuable in swabbing out abscess cavities. Impaction of the third stomach and cornstalk disease are frequent and the fatality great. The symptoms of these diseases appear to me very similar at times, and treatment must be entirely preventive. Glanders and farcy are met with occasionally. Malignant catarrh of the ox has been epizoötic throughout the northeastern portion of the State, but was of a rather mild type and readily amenable to ordinary remedies.

In two herds of cattle in Floyd County afflicted with contagious abortion I applied treatment which consisted of the daily application of antiseptic washes to the external genitals, rump, and quarters; thorough disinfection of the stable and discharges; careful removal of the membranes when retained, and irrigation of the uterus. In addition, 4 drachms of a 3 per cent. solution of carbolic acid were given subcutaneously once daily for the first week; then the dose was gradually decreased until at the expiration of a month it was discontinued. The disinfection was kept up a month longer. After that time all signs of abortion had disappeared completely from the two herds.

Dr. J. G. Parslow writes that in his territory, during May and June, epizoötic cellulitis prevailed extensively in severe form, but caused but little loss of life.

Blackleg caused considerable anxiety in sections, and is on the increase, owing to lack of confidence in vaccination on the part of the farmers. Cornstalk disease has caused considerable loss. It appeared from two to four weeks after the cattle were turned into the fields. No remedy seems of any avail. Dr. P. O. Koto reports cornstalk disease in his part of the State. Dr. H. E. Talbot writes that cornstalk disease has been very prevalent and very fatal. He has also had about 200 cases of what is similar to foot-and-mouth disease. The disease has all the symptoms of contagious aphtha, and is as much like what we have been taught to call foot-and-mouth disease as a twin brother, and may be called that for want of a better name.

J. H. McLeod,
Chairman.

On motion, the Association went into secret session.

Dr. Heck moved that the Secretary be instructed to invite only graduated veterinarians to our next meeting. Seconded, voted upon, and carried.

On motion, the Association adjourned.

The following members were in attendance at the meeting: G. Lames, Dysart; C. A. Clinton, Havelock; George M. Walrod, Storm Lake; C. E. Stewart, Chariton; John J. Repp, Ames; P. O. Koto, Forest City; W. A. Heck, Maquoketa; P. Malcolm, New Hampton; A. B. Wilmoth, Des Moines; A. A. Adamson, Newton; W. H. Austin, Newton; John H. McNeall, Ames; S. H. Kingery, Creston; D. E. Baughman, Fort Dodge; M. Y. Schaffer, Des Moines; S. H. Bauman, Birmingham; F. J. Neiman, Marshalltown; S. T. Miller, Shelby; S. K. Hazlet, Oelwein; D. H. Miller, Harlan; H. C. Simpson, Denison; H. E. Talbot, Des Moines; H. L. Stewart, Lacona; Joseph Biggs, Union; T. A. Shipley, Cedar Rapids; J. I. Gibson, Denison; J. A. Campbell, Des Moines; George A. Scott, Independence; J. R. Sanders, Corydon; N. A. Kippen, Riceville; J. S. Potter, Iowa City; G. L. Buffington, Baxter, and C. W. Stevens, Knoxville.

The following visitors were in attendance: Hon. W. M. Greeley, Ames; Drs. C. C. Lyford, Minneapolis; S. D. Brimhall, Minneapolis, Minnesota; Thomas D. Hulme, Commerce; Carl W. Gay, Ames; H. B. Treman, Sioux City; W. L. Evers, Iowa Falls; Messrs. C. E. Harlan, Des Moines; W. J. Wallace, Des Moines; C. G. Martin, Des Moines; A. C. Lookingbill, Yale; Ira W. Edwards, Redfield; P. C. Price, Shell Rock; F. A. Blake, Harper;

Zan Cotter, Chicago, Ill; A. W. Russell, Meservey; A. F. Baldwin, Ames; Walter E. Miller, Ames; Albert Stigers, Stuart; J. C. Boyd, Kansas City; R. H. Stevenson, Sigourney; Wm. R. Simpson, Elliott; H. M. Stevenson, Perry; H. C. Dillman, Oakley; J. E. Harley, Chicago; J. N. Cozzens, Col.; S. T. Bodell, Winthrop; W. L. Turner, New Hampton.

Respectfully submitted,
JOHN J. REPP,
Secretary.

